# Mechanical and Damping Properties of Recycled Aggregate Concrete Modified with Air-Entraining Agent and Polypropylene Fiber

**DOI:** 10.3390/ma13082004

**Published:** 2020-04-24

**Authors:** Chonggang Zhou, Xingwang Pei, Wenlong Li, Yijun Liu

**Affiliations:** College of Civil Engineering, Xi’an University of Architecture & Technology, Xi’an 710055, China; wisdom181@126.com (C.Z.); li1536295967@126.com (W.L.); appleliuyijun@sina.com (Y.L.)

**Keywords:** recycled aggregate concrete, interface transition zone, static properties, dynamic behavior, damping mechanism, air-entraining agent

## Abstract

In this study, recycled aggregate concrete (RAC) modified with polypropylene fiber (PP) and air-entraining agent (AGA) was prepared, and the effects of PP and AGA on the static (compressive strength, Young’s modulus, and splitting tensile strength) and dynamic properties (dynamic modulus of elasticity and damping ratio) of RAC were investigated. The experimental results showed that the addition of an AGA and PP had a favorable effect on the damping ratio of the concrete, however, the addition of the AGA had a slightly negative effect on the mechanical performance of the concrete. The AGA and PP contents required to achieve the optimum damping ratio of the concrete with the least reduction in the mechanical performance were 0.02% and 0.10%, respectively. Furthermore, the addition of AGA was more effective than that of PP in improving the damping property of the concrete.

## 1. Introduction

Concrete is the most common construction material and has been extensively used in buildings, bridges, and dams owing to the advantages of its low cost, abundant raw materials, high strength, and excellent durability [1,2]. Aggregate accounts for approximately 60–70% of all raw materials in the concrete production [3,4,5,6]. However, concrete consumption is expected to increase with the continuous growth of the infrastructure industry [7,8]. Consequently, non-renewable aggregate resources may get exhausted [4]. Therefore, an alternative material to replace natural aggregate and to relieve the current pressure on the sustainable development of environment is urgently needed [4,9].

Recycled concrete aggregate (RCA) is manufactured from abandoned concrete blocks by a series of processes, including washing, crushing, and grading. RCA is a renewable resource and can be used to alleviate environment pollution [10,11]. Studies have indicated that the partial replacement of natural concrete aggregate (NCA) with RCA to prepare recycled aggregate concrete (RAC) has immense potential for practical applications [12]. RAC is composed of the original aggregate, an adhered old cement mortar, and two types of interface transition zones (ITZs) [4]. However, it exhibits weaker mechanical properties and lower durability than NCA due to the inferior properties of its constituents [4,12].

Dynamic characteristics govern the intrinsic properties of a material and can reflect the behavior of a material under dynamic loading [13]. Such characteristics facilitate the dispersion and conversion of energy during the vibration process [7,14]. Further, they can help in improving the stability and safety of the concrete structure. There are three types of damping in concrete [7]: system damping, structural damping, and material damping. System and structural damping are external damping (viscous damping, dry damping, hysteresis damping, nonlinear damping, etc.) [15,16], and material damping is internal damping (medium damping, friction damping, etc.) [17]. The damping ratio is the dominant property that indicates the dynamic characteristics of a material [18,19,20]. Therefore, it has been widely used for the dynamic analysis of building structures [4,7,8,9,10]. To date, several studies have focused on the damping of RAC [11], while some have mainly focused on the effect of the replacement ratio and size of RCA on the damping performance of RAC [4,7].

Studies have suggested that due to its high tensile strain, a fiber polymer, as an additive, can contribute to improving the performance of concrete [3,7,21,22]. Furthermore, the study of the effect of the type and dosage of fiber on the mechanical properties of RAC has shown that concrete reinforced with glass and steel fibers can significantly improve the mechanical performance of RAC when the fiber content is within a reasonable range [23]. Additionally, the air entraining agent (AGA) introduces many bubbles in concrete, and it may increase the damping performance of the concrete to a certain extent. Few studies have investigated the properties of RAC modified with polypropylene fiber (PP) and AGA. Specifically, the damping properties of pure concrete and concrete modified with AGA and PP have rarely been compared.

In this study, for the first time, RAC was incorporated with the advantage of PP as well as the high damping characteristic of AGA. The damping properties of RAC at various PP and AGA contents were compared with those of the reference concrete. Our results provide a deep understanding of the dynamic behavior of RAC modified with PP and AGA and potentially serve as an effective tool for designing concrete with excellent damping property.

## 2. Materials and Methods

### 2.1. Raw Materials

#### 2.1.1. Cementitious Materials

**Figure 1:**
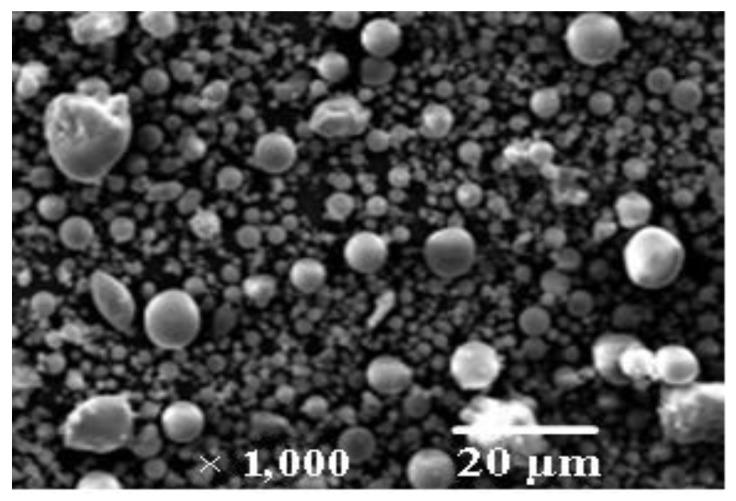
Scanning electron micrograph of fly ash (FA).

**Figure 2:**
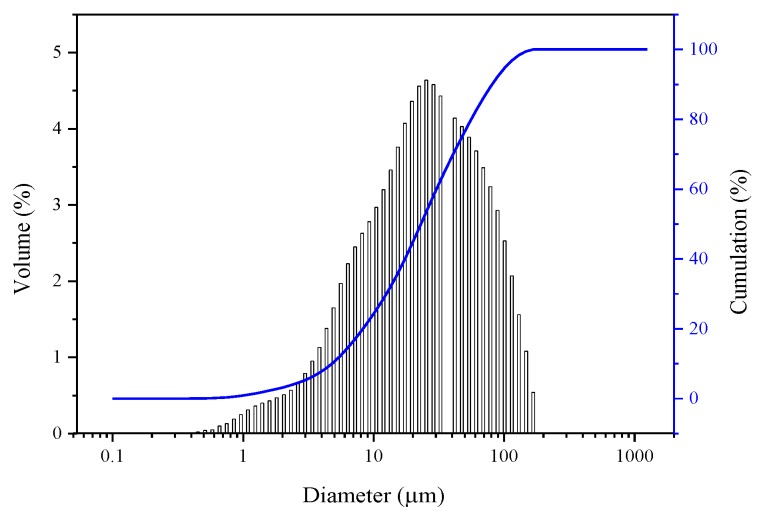
Particle size distribution of FA.

The cementitious materials used were ordinary Portland cement (P·O 42.5) and fly ash (FA). The cement was purchased from Shaanxi Qinling Cement Co., Ltd. (Yaoxian, Shaanxi, China), and the FA was purchased from Shenmu County Huatai Clean Coal Technology Development Co., Ltd. (Shenmu, Shaanxi, China). Table 1 presents the chemical composition and physical properties of cement according to Chinese Standard GB 175-2007 [24]. Table 2 shows the chemical composition of FA, which complies with the requirements of the Chinese Standard GB/T 51003-2014 [25]. Figure 1 and Figure 2 show the scanning electron micrograph and particle size distribution of FA, respectively.

#### 2.1.2. Aggregate

The fine aggregate used was river sand (RS) with a fineness modulus of 2.60 (medium sand); it has water absorption of 1.15% and specific gravity of 2.45. The coarse aggregates used were NCA (broken granite gravel) and RCA (laboratory waste concrete block) and with continuous grading. The maximum size of the coarse aggregates was 25 mm. Table 3 lists the properties of RCA and NCA. The properties of fine and coarse aggregates complied with the Chinese standards GB/T 14684-2011 [26] and GB/T14685-2011 [27], respectively.

#### 2.1.3. Polypropylene Fiber

PP with a length of 12 mm was used to improve the damping property of RAC. Table 4 shows the primary physical and mechanical properties of PP.

#### 2.1.4. Admixture

Polycarboxylate superplasticizer (SP) with a water reduction ratio of 30% and solid content of 35% was used in this study. An AGA with a specific density of 1.02% was used to enhance the damping property of the concrete.

### 2.2. Mixture Preparation

Nine different mixtures of RAC modified with AGA (wt.%) and PP (V%) were prepared. Table 5 and Table 6 show the mixture proportions of reference concrete and RAC, respectively. The specimens were formed based on the content. For e.g., in 50RAC-A1, 50 indicates that the replacement percentage of RCA is 50% (wt.), and A1 indicates that the content of AGA is 0.072 kg/m^3^. The mixing water consisted of two parts: water consumption of ordinary concrete mix design and additional water. It should be noted that the additional water did not change the effective water-to-binder ratio of the mixture. 

### 2.3. Test Methods

For testing the compressive strength, Young’s modulus, and splitting tensile strength, 150 mm × 150 mm × 150 mm cubic molds, 100 mm × 100 mm × 400 mm prism, and 150 mm × 150 mm × 300 mm prism cubic molds were used, respectively, according to GB/T 50081-2002 [28]. The test samples were demolded 24 h after the initial casting and were put in a standard curing room (20 ± 1 ℃, ≥95% RH) until the testing day.

The dynamic modulus of elasticity of the concrete was determined by the resonant frequent method in accordance with GB/T 50082-2009 [29]. It was calculated according to the following equation:

(1) $$E_{d}=13.244\times 10^{-4}\times \frac{WL^{3}f^{2}}{\alpha^{4}}$$

where *E_d_*, W, L, *f*, and α represent the dynamic modulus of elasticity of RAC (MPa), weight of sample (kg), length of test sample (mm), transverse frequency of the specimen (Hz), and the side length of square cross-section sample (mm), respectively.

**Figure 3:**
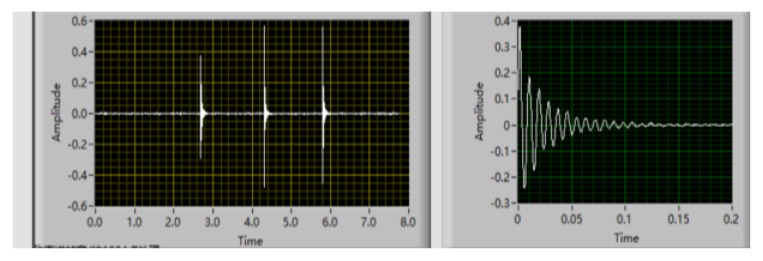
Time-magnitude signal as the acceleration response signal.

**Figure 4:**
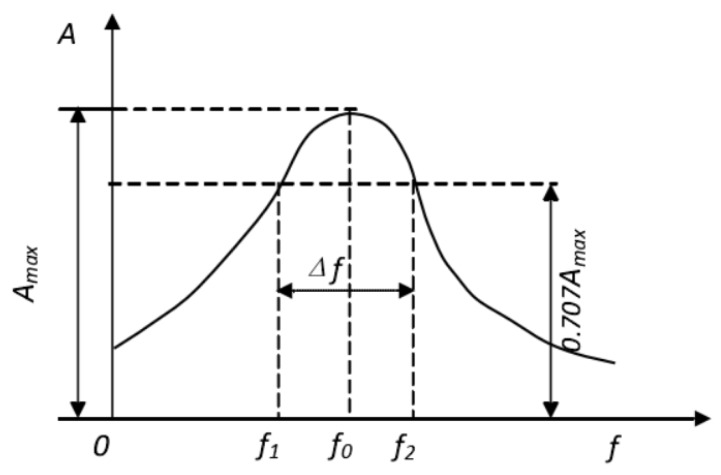
Estimation of the damping ratio of concrete using the half-power bandwidth method [29].

The damping ratio of concrete was evaluated by the vibration of a free-free beam. A pulse hardware/software vibration analysis system (Brüel and Kjær) was utilized to record the acceleration response signals as time magnitude and hammer force, as shown in Figure 3. The ME’Scope modal analysis program was used to record the data and convert it to frequency–magnitude (m^2^/s) graphs, as shown in Figure 4, and then the resonant frequency (f_0_) was obtained [7]. The half-power bandwidth method was applied to measure the damping ratio of the concrete [30]. All specimens with a dimension of 100 mm × 100 mm × 400 mm underwent damping ratio test after 28 days of curing. The damping ratio of concrete can be expressed as follows:

(2) $$\xi =\frac{\left(f_{1}-f_{2}\right)}{2f_{0}}$$

where *ξ*, f_1_, and f_2_ represent the damping ratio of concrete (%), frequency corresponding to an amplitude of f_0_ /√2 (Hz), and the resonant frequency of concrete (Hz), respectively.

## 3. Results and Discussion

### 3.1. Static Properties

#### 3.1.1. Compressive Strength

**Figure 5:**
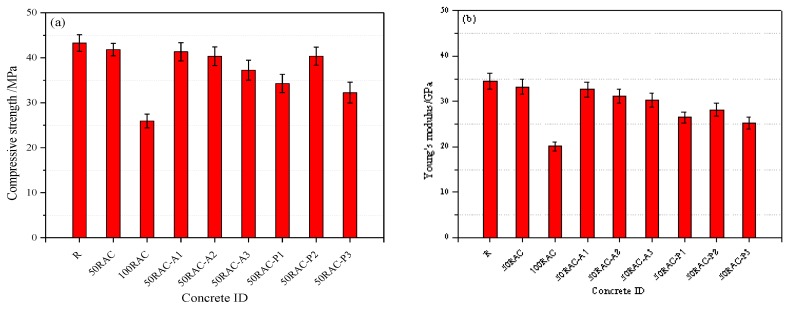
Static properties of concrete after 28 days of curing: (**a**) compressive strength and (**b**) Young’s modulus.

Figure 5a shows the compressive strength of the reference concrete and RAC modified with PP and AGA. The compressive strength of the samples decreased by 3.37% (50RAC) and 40.04% (100 RAC) as compared to that of the reference concrete after 28 days of curing. It is evident that RCA has a marginal effect on the compressive strength of RAC with replacement percentage of 50%, while the RAC with replacement percentage of 100% is significantly affected. Similar results were reported by Liang et al. [4]. This behavior can be attributed to the higher absorption and crushing value of RCA, as shown in Table 3.

Further, a large number of micro-cracks and voids exist in the old cement mortar and ITZs of RAC, as seen in Figure 5a. The accumulation of water film at the ITZ near the surface of the aggregate and the growth of a large amount of hydration products, calcium hydroxide and ettringite (CH and AFt) on the surface of ITZ cause a porous ITZ in the RAC [4]. Furthermore, RAC has weak ITZs due to the poor adherence between the old aggregate and the new cement mortar, which causes the specimen (100RAC) to exhibit the lowest compressive strength [11,12]. Moreover, the weak properties of RCA (Table 3) and the characteristics of ITZs positively affected the energy dissipation of RAC. This is discussed in Section 3.2.2.

The compressive strength of RAC slightly decreased by 1.15% (50RAC-A1) and 3.54% (50RAC-A2) when the AGA content was below 0.02%, but was sharply reduced to 10.95% (50RAC-A3) for the mixture containing 0.03% AGA as compared to that of the 50RAC sample after 28 days of curing. This could be attributed to the increment of porosity due to the introduction of AGA in concrete.

**Figure 6:**
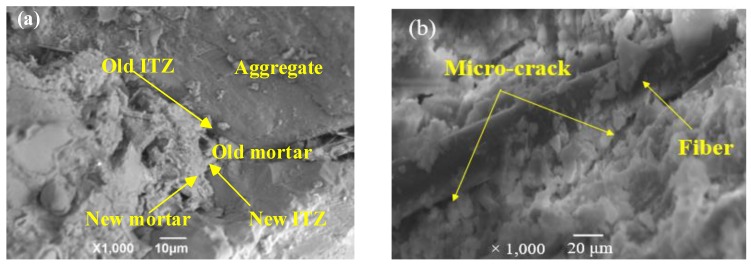
Interface transition zones (ITZs) in RAC: (**a**) ITZ between aggregate and cement mortar and (**b**) ITZ between fiber and cement mortar.

The compressive strength of the RAC decreased by 18.03% (50RAC-P1), 3.15% (50RAC-P2), and 22.89% (50RAC-P3) than that of 50RAC after 28 days of curing. The PP-reinforced RAC consisted of six phases: original aggregate, natural aggregate, cement mortar (new cement mortar and old cement mortar), new ITZ (between the NCA and new cement mortar), old ITZ (between the NCA and old cement mortar), and ITZ between the fiber and cement mortar [4]. A large number of micro-cracks were observed in the ITZ between the fibers and cement mortar, as shown in Figure 6. In addition, the thickness of the fiber-cement mortar ITZ was larger than that of the old ITZ in RAC, resulting in a significant gap between the micro-hardness of ITZs [1,10]. This hindered the mechanical performance of RAC.

#### 3.1.2. Young’s Modulus

Figure 5b shows the Young’s modulus of the reference concrete and the RAC modified with PP and AGA. It is evident that the effect of RCA on the reduction of Young’s modulus is stronger than that of the addition of AGA and PP.

The Young’s modulus of the RAC decreases by 3.77% and 41.74% for RCA with replacement percentage of 50% and 100%, respectively, as compared to that of the reference concrete. Furthermore, the Young’s modulus of the RAC decreased with increasing AGA content. A slightly decreasing trend is observed when the AGA content is less than 0.02%. However, when the AGA content is higher than 0.02%, the Young’s modulus of the specimens (50RAC-A3) exhibit a significant reduction. Figure 5b shows that the Young’s modulus of the mixtures 50RAC-P1, 50RAC-P2, and 50RAC-P3 containing 0.09%, 0.10%, and 0.11% PP is reduced by 15.52%, 10.15%, and 25.07%, respectively, as compared to that of 50RAC. 

Previous studies have indicated that the properties of aggregates significantly affect the Young’s modulus of concrete [3,4]. The reduction in Young’s modulus is primarily attributed to the low strength and elastic modulus of RCA. Therefore, the Young’s modulus of concrete can be significantly controlled by the properties of RCA.

#### 3.1.3. Splitting Tensile Strength

**Figure 7:**
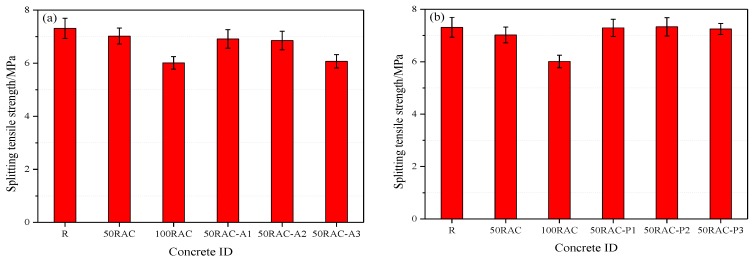
Splitting tensile strength of concrete after 28 days of curing: (**a**) reference concrete and RAC modified with AGA and (**b**) reference concrete and RAC modified with PP.

Figure 7 shows the splitting tensile strength of the reference concrete and RAC modified with PP and AGA. The effect of RCA on the splitting tensile strength of RCA is similar to that on the compressive strength. The splitting tensile strength of the concrete decreases by 3.97% (50RAC) and 17.78% (100RAC) as compared to that of the natural aggregate concrete (NAC) after 28 days of curing.

Furthermore, the splitting tensile strength of concrete decreases by 1.57% (50RAC-A1), 2.42% (50RAC-A2), and 13.53% (50RAC-A3) as compared to that of the 50RAC samples. Therefore, a similar conclusion as that from the compressive strength test can be drawn, i.e., a higher dose of AGA adversely affects the splitting tensile property of the RACs regardless of the replacement ratio of the RCA. While a slight increment was found for the specimen containing PP, the splitting tensile strength of concrete increased by 3.85% (50RAC-P1), 4.42% (50RAC-P2), and 3.27% (50RAC-P3). Similar conclusions have been reported by earlier studies [23,31].

### 3.2. Dynamic Behavior

**Figure 8:**
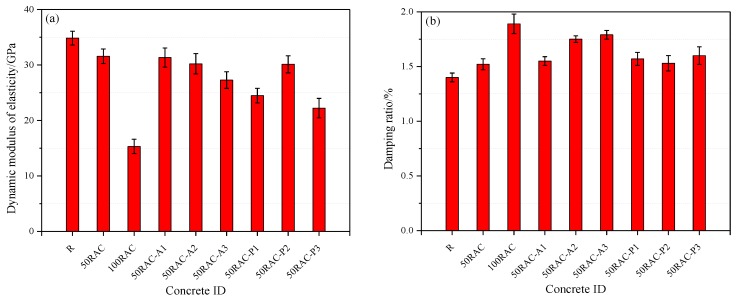
Dynamic behavior of the reference concrete and RAC modified with AGA and PP: (**a**) dynamic modulus of elasticity and (**b**) damping ratio.

The dynamic behavior, i.e., the dynamic modulus of elasticity and damping ratio of the reference concrete and the RAC modified with AGA and PP, is shown in Figure 8.

#### 3.2.1. Dynamic Modulus of Elasticity

It is evident from Figure 8a that the dynamic modulus of elasticity of the RAC decreases by 9.38% (50RAC) and 56.07% (100RAC) as compared to the reference concrete.

The dynamic modulus of elasticity of the samples 50RAC-A1, 50RAC-A2, and 50RAC-A3 decreases by 1.60%, 4.34%, and 13.65%, respectively, as compared to that of 50RAC specimen. It is clear seen that the dynamic modulus of elasticity decreases with the increase in the replacement percentage of RCA and the AGA content. The dynamic modulus of elasticity of the samples 50RAC-P1, 50RAC-P2, and 50RAC-P3 decreases by 22.51%, 4.62%, and 29.64%, respectively, indicating that the addition of PP can significantly reduce the dynamic modulus of elasticity of RAC with the exception for concrete with 0.50% PP.

#### 3.2.2. Damping Ratio

The damping ratio of concrete exhibits a sharply increasing trend with the increase in the replacement percentage of RCA, as seen in Figure 8b. The damping ratio of the samples 50RAC and 100RAC is enhanced by 7.86% and 35.00%, respectively, as compared to that of the reference concrete after 28 days of curing. Similar results have been reported by Liang et al. [4], Lin et al. [10], and Li et al. [11]. The elastic modulus of different phases significantly varies in RAC. For e.g., the elastic modulus of the aggregate is much larger than that of the cement mortar and ITZs (old ITZ and new ITZ). Further, there is a considerable difference between the elastic modulus of various phases [4]. 

**Figure 9:**
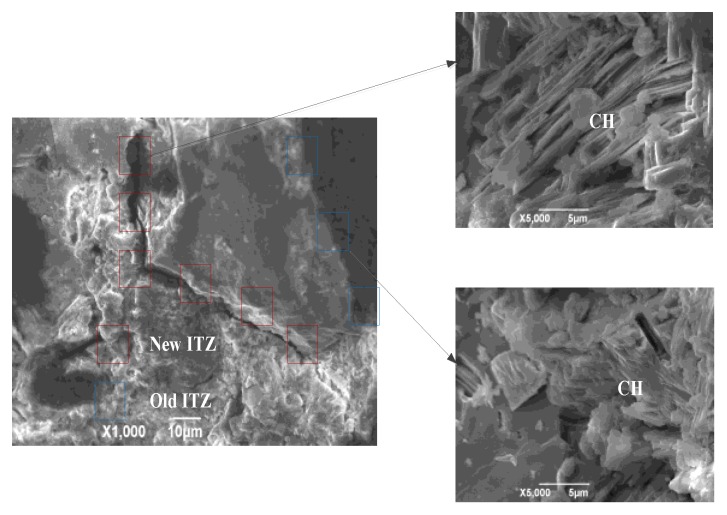
Images showing ITZs in RAC.

Previous reports have indicated that the indentation modulus of new cement mortar is 1.18 times that of new ITZ, while the indentation modulus of the old cement mortar is approximately 1.33 times higher than that of the old ITZ in the RAC [4], which is confirmed in Figure 9. Hence, the addition of RCA can increase the proportion of the old cement mortar and ITZs of RAC, which enhances the inhomogeneity of RAC and contribute to the relative deformation between different phases in RAC [11,30,31].

**Figure 10:**
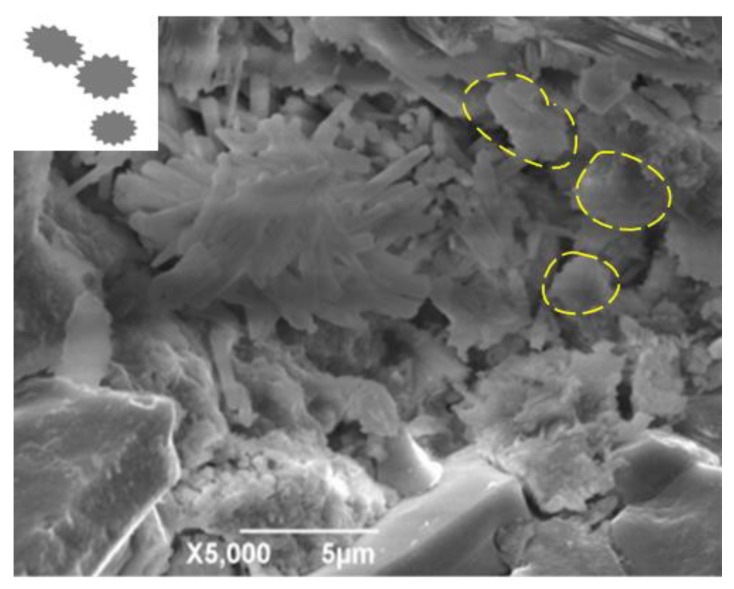
Bubbles and voids in RAC.

The damping ratio of RAC increases with the increase in the AGA content. This increasing trend is more obvious when the AGA content is above 0.02%. The central function of the AGA is to introduce several air bubbles and pores into the concrete, which plays a vital role in flexible cushioning bag when the structure is subjected to external vibration, as shown in Figure 10. The bubbles and voids in the RAC improve the damping capacity of RAC. As the AGA content increases, the introduction of bubbles and voids reaches a certain amount, and the damping property of the RAC attains its optimal value. Further increase in the amount of AGA does not have a significant effect on damping ratio of RAC. On the contrary, it significantly reduces the mechanical properties of the RAC, as discussed in Section 3.1. Therefore, for the same replacement percentage of RAC, the optimal content of AGA to simultaneously attain improved mechanical properties and dynamic characteristics is 0.02%.

The damping ratio of the samples 50RAC-P1, 50RAC-P2, and 50RAC-P3 increases by 3.18%, 0.66%, and 5.26%, respectively, as compared to that of the 50RAC specimen after 28 days of curing. This can be attributed to the significant gap between the micro-hardness of ITZs (new ITZ, old ITZ, and fiber-cement mortar ITZ), which increases the energy dissipation of RAC due to the sliding between the ITZs [18], as shown in Figure 9. The proportion of ITZs increases with the content of PP, which leads to higher energy dissipation of RAC. All these effects are conducive to improve the damping ratio of RAC. On the contrary, the bubbles and voids in the RAC adversely affect the mechanical properties of RAC, as discussed in Section 3.1. Therefore, it can be concluded that the addition of AGA is more effective than that of PP for increasing the damping ratio of RAC.

## 4. Conclusions

We prepared RAC containing PP and AGA and evaluated their damping properties and mechanical performance at various AGA and PP contents. The important results of this study are summarized as follows:

1. The RCA had a marginal effect on the mechanical properties of RAC with a replacement aggregate percentage of 50% (reduced by approximately 5%). However, the mechanical properties of the RAC with 100% replacement aggregate were significantly affected (approximately decreased by 40%) than those of the reference concrete.
2. The addition of both AGA and PP adversely affected the mechanical properties of the concrete, and the effect of PP addition on the reduction of the mechanical properties of RAC was stronger than that of AGA addition. The compressive strength and Young’s modulus reduced slightly when the AGA content was below 0.02%.
3. The addition of both AGA and PP favorably affected the damping property of the concrete, and the addition of AGA was more effective than that of PP in increasing the damping ratio of the RAC. Furthermore, the AGA and PP contents to achieve the optimum dynamic property of the concrete with the least reduction in the mechanical performance were determined to be 0.02% and 0.10%, respectively.

## Figures and Tables

**Table 1:** Chemical composition and physical properties of the cement.

Chemical Composition (wt.%)								Primary Physical Properties		
CaO	SiO_2_	Al_2_O_3_	Fe_2_O_3_	SO_3_	MgO	Na_2_O	K_2_O	Specific Surface Area (m^2^/kg)	Specific Gravity (kg/m^3^)	LOI (Loss on Ignition, %)
61.89	18.73	5.88	3.37	2.08	3.24	0.31	0.15	360	3170	3.03

**Table 2:** Chemical composition of the FA (wt.%).

SiO_2_	Al_2_O_3_	Fe_2_O_3_	CaO	K_2_O	Na_2_O	MgO	SO_3_	ZnO	TiO_2_	P_2_O_5_
49.72	31.09	6.04	3.81	0.41	0.17	1.53	1.49	0.08	2.58	0.42

**Table 3:** Properties of coarse aggregate.

Type	Water Absorption (%)	Crushing Value Index (%)	Apparent Density (kg/m^3^)	Bulk Density (kg/m^3^)
NCA	1.06	9.80	2561	1380
RCA	5.48	15.7	2509	1253

**Table 4:** Main physical and mechanical properties of polypropylene fiber (PP).

Diameter (µm)	Density (kg/m^3^)	Tensile Strength (MPa)	Elastic Modulus (GPa)	Elongation (%)
15–40	920	450	3.81	20

**Table 5:** Mixture proportion of reference concrete (kg/m^3^).

Concrete ID	Cement	FA	RS	NCA	RCA	Water (Additional Water)	PP	SP	AGA
R	280	70	640	1040	0	175 (0)	0	3.5	0

**Table 6:** Mixture proportions of reference concrete and recycled aggregate concrete (RAC).

Concrete ID	NCA (%)	RCA (%)	PP (%)	AGA (%)	SP(%)	Water (Additional Water) (kg/m^3^)
R	100	0	0	0	1	175 (0)
50RAC	50	50	0	0	1	175 (23.2)
100RAC	0	100	0	0	1	175 (46.4)
50RAC-A1	50	50	0	0.01	1	175 (23.2)
50RAC-A2	50	50	0	0.02	1	175 (23.2)
50RAC-A3	50	50	0	0.03	1	175 (23.2)
50RAC-P1	50	50	0.1	0	1	175 (23.2)
50RAC-P2	50	50	0.5	0	1	175 (23.2)
50RAC-P3	50	50	1.0	0	1	175 (23.2)
